# Exploring the role of miRNAs in Hepatocellular Carcinoma: Insights into signaling pathways and their regulatory mechanisms

**DOI:** 10.12669/pjms.41.5.11444

**Published:** 2025-05

**Authors:** Necip Altundas, Eda Balkan, Murat Kizilkaya, Elif Demirci

**Affiliations:** 1 Necip Altundas Department of General Surgery, Ataturk University, 25240 Erzurum, Turkey; 2 Eda Balkan Department of Medical Biology, Ataturk University, 25240 Erzurum, Turkey; 3 Murat Kizilkaya Department of Medical Biology, Ataturk University, 25240 Erzurum, Turkey; 4 Elif Demirci Department of Pathology, Ataturk University, 25240 Erzurum, Turkey

**Keywords:** microRNAs, Hepatocellular carcinoma, Non-coding RNAs

## Abstract

**Objective:**

This prospective case-control study aimed to assess the predictive value of the expression levels of Hsa-miR-200-3p, Hsa-miR-155-5p, Hsa-miR-20a-5p, Hsa-miR-221-3p, Hsa-miR-34a-5p, Hsa-let-7b-5p, and Hsa-miR-125b-5p microRNAs (miRNAs) in the diagnosis, prognosis, and treatment response of hepatocellular carcinoma (HCC) patients. The study includes 20 healthy controls and 25 HCC patients.

**Methods:**

The study was designed as a prospective case-control study and was conducted between 2023 to 2024 at Ataturk University, in collaboration with the Departments of Organ Transplantation, Medical Biology and Medical Pathology. miRNA expression levels were examined in HCC tissue samples. RNA was extracted from paraffin-embedded tissues, and miRNA expression levels were measured using reverse transcription-quantitative PCR. The relationships between clinical factors and miRNA expression levels were assessed.

**Results:**

Significant differences in expression levels were found between HCC tissues and healthy control tissues for Hsa-miR-20a-5p, Hsa-miR-125b-5p, and Hsa-let-7b-5p. A significant correlation was observed between Hsa-miR-34a-5p and γ-glutamyl transferase levels (P<0.05), as well as between Hsa-miR-20a-5p and albumin levels (P<0.05). Significant differences in expression levels were found between patients with and without HBV infection for let-7b-5p (P=0.007) and miR-200-3p (P=0.013).

**Conclusion:**

This study demonstrates that miRNAs, particularly Hsa-miR-20a-5p and Hsa-miR-34a-5p, may regulate the Wnt/β-catenin pathway and influence the progression of HCC. Disease progression was assessed by comparing miRNA expression levels with clinical markers (such as γ-glutamyl transferase and albumin). High expression of Hsa-miR-20a-5p affects the Wnt/β-catenin pathway and plays a significant role in disease processes. Additionally, the correlation between Hsa-miR-34a-5p and γ-glutamyl transferase indicates disease progression. However, further advanced studies are required to better understand how the Wnt/β-catenin pathway is regulated by miRNAs and the impact of this regulation on the disease.

## INTRODUCTION

Hepatocellular carcinoma (HCC) is the third most common cause of cancer-related deaths globally, accounting for 70–85% of cases^1^,^2^ More than 90% of HCC is caused by viral hepatitis, alcohol abuse, or non-alcoholic fatty liver disease.^2^ Early-stage HCC is usually treated with curative approaches such as resection, transplantation and ablation.^3^ Dysregulated signaling pathways, such as the Wnt/β-catenin signaling pathway, play an important role in HCC progression. This pathway is constantly activated by genetic and epigenetic changes and supports tumor growth.^4^,^5^ MicroRNAs (miRNAs) are small non-coding RNAs that regulate gene expression at the post-transcriptional level. miRNAs can function as oncogenes or tumor suppressors depending on their target genes.^6^ This study examined how the Wnt/β-catenin pathway is regulated by miRNAs in HCC and the contribution of this regulation to disease progression. The findings aim to understand the effects of miRNAs on this pathway, providing potential biomarkers or treatment targets that can be used in HCC diagnosis and prognosis.

## METHODS

This prospective observational study was conducted in 2023 to 2024 at Ataturk University Yakutiye Hospital. It included 25 primary hepatocellular carcinoma (HCC) patients and 20 healthy controls. (The healthy control group was thoroughly assessed before being included in the study, and no pathological findings related to liver disease were observed. This group consisted of healthy. HCC diagnosis was based on histopathology and clinical findings. The patient group (84% men, 16% women) had a median age of 65 years, while the control group (65% men, 35% women) had a median age of 61 years.

### Ethical Approval:

It was obtained form the institutional ethics committee Ref. B.30.2.ATA.0.01.7-13:738-2023; Dated: October 26, 2023

### Sampling and Histological Examination:

Formalin-fixed, paraffin-embedded (FFPE) liver biopsies were examined histopathologically and stained with hematoxylin-eosin (H&E). Immunohistochemistry (IHC) analysis using Hep Par-1 was performed to evaluate liver cell encapsulation.

### miRNA Expression Analysis:

RNA was isolated from FFPE samples using the miRNeasy FFPE Kit (Qiagen) and validated via NanoDrop. Quantitative reverse transcription PCR (qRT-PCR) Kit (Qiagen) was used to assess miRNA levels related to the Wnt/β-catenin pathway, including Hsa-miR-200c-3p, Hsa-miR-155-5p, Hsa-miR-20a-5p, Hsa-miR-221-3p, Hsa-miR-34a-5p, Hsa-let-7b-5p, and Hsa-miR-125b-5p. Expression levels were compared with the control group, and statistical significance was evaluated.

### Statistical Analysis:

Data were analyzed using SPSS 24.0. Continuous variables were presented as mean ± standard deviation, while categorical variables were shown as frequencies. Group differences were analyzed with t-tests or Mann-Whitney U tests. A p-value <0.05 was considered significant. ROC curves and regression analyses were used to assess predictive value, with AUC calculated to determine diagnostic accuracy.

## RESULTS

### Demographic and Clinical Characteristics:

About 84% of the patients were male and 16% were female; the median age was 63 years (ranging from 40 to 81 years). In the control group, these ratios were 65% and 35%, respectively.

### Biochemical and Hematological Findings:

The median AFP level in the patients was 68 ng/mL, ALT 16.9 U/L, AST 21.8 U/L, GGT 18.4 U/L, ALP 126.4 U/L, total bilirubin 0.87 mg/dL, and CRP 7.33 mg/L. The NLR (Neutrophil-to-Lymphocyte Ratio) was 6.44 and the PLR (Platelet-to-Lymphocyte Ratio) was 177.77, both significantly higher compared to the control group (P < 0.05).

### Staging and Imaging:

About *44*% of the patients met the Milan criteria. 76% underwent MRI, and 88% had CT scans; 68% of the patients had a single nodule, while 8% had multiple nodules. No portal vein thrombosis was observed.

### Transplantation and Lifestyle:

**Fig.1:**
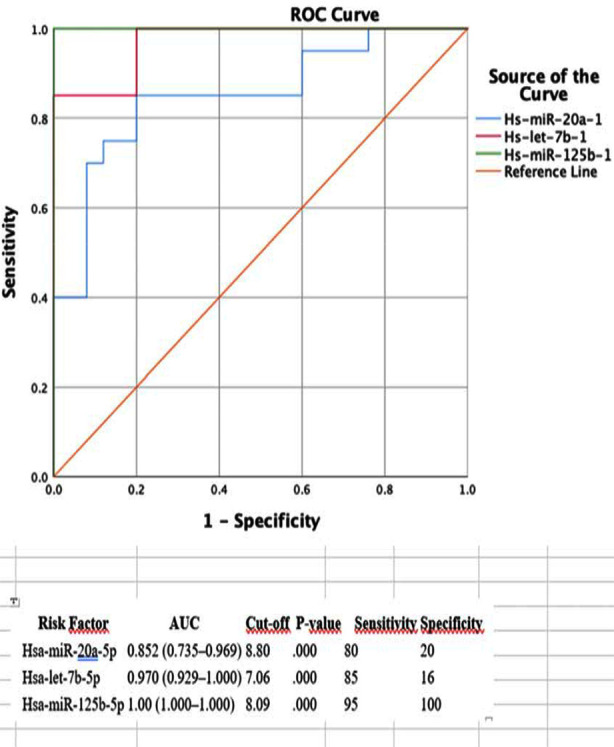
ROC Analysis Results: AUC Values of miRNA.

RT-qPCR was performed on all tissue samples, demonstrating a significant increase in the expression of Hsa-miR-20a-5p, Hsa-let-7b-5p, and Hsa-miR-125b-5p compared to healthy tissues (P<0.05). However, no significant difference was observed between the two groups for Hsa-miR-155, Hsa-miR-221-3p, and Hsa-miR-34a-5p (Table-I). ROC analysis is shown in Fig.1.

**Table-I:** miRNA expression levels in patients with HCC compared with the control group.

miRNA	Average, ΔCq	Average, ΔCq	2^-ΔΔCq^	2^-ΔΔCq^	P-value	Fold change
	Control group	Patient group	Control Group	Patient Group		
Hsa-miR-200c-3p	-0.47	0.46	1.384630	0.724772	>0.090	-1.91
Hsa-miR-155-5p	0.83	1.72	0.561361	0.302708	>0.056	-1.85
Hsa-miR-20a-5p	0.74	1.81	0.597496	0.284322	<0.002	-2.10
Hsa-miR-221-3p	1.62	1.84	0.326239	0.279322	>0.084	-1.17
Hsa-miR-34a-5p	1.65	1.73	0.319636	0.301786	>0.067	-1.06
Hsa-let-7b-5p	-1.77	0.69	3.401097	0.618480	<0.034	-5.50
Hsa-miR-125b-5p	-1.04	1.96	2.061222	0.256672	<0.002	-8.03

### Univariate and Multiple Logistic Regression Analysis of Factors Associated with Patient and Control Groups:

Association between risk variables and HCC. Binary logistic regression analysis was used to analyze the risk factors influencing the disease. Based on the univariate model, increasing the ALP value by one unit increased the risk of disease by 1.076-times (P=0.002); increasing the total bilirubin value by one unit increased the risk of disease by 15.653-times (P=0.016); increasing the neutrophil value by one unit increased the risk of disease by 1.918-times (P=0.006); and increasing the lymphocyte parameter decreased the risk of disease (odds ratio=0.307; P=0.018 (Table-III).

**Table-II:** Association between laboratory results and miRNA expression in patients with hepatocellular carcinoma.

Variable	Hsa-SNORD61	Hsa-miR-200c-3p	Hsa-miR-155-5p	Hsa-miR-20a-5p	Hps-miR-22-3p1	Hsa-miR-34a-5p	Hsa-let-7b-5p	Hsa-miR-125b-5p
HBV								
Negative								
Mean ± SD	9.61±1.09	7.23±1.12	9.49±0.9	10.58±1.14	10.27±1.28	9.99±0.96	7.58±1.04	9.99±0.94
Median (min-max)	9.49 (8.31-11.5)	7.23 (5.7-9.13)	9.29 (8.61-11.34)	10.78 (8.77-12.43)	9.99 (8.86-12.27)	9.93 (8.48-11.64)	7.45 (6.19-9.47)	9.67 (8.74-11.7)
Positive								
Mean ± SD	8.08±3.06	9.88±2.66	10.67±2.21	10.29±2.89	10.47±2.56	10.44±2.26	10.06±2.24	10.79±1.26
Median (min-max)	8.11 (4.02-16.71)	10.15 (4.49-16.12)	10.44 (7.92-17.53)	9.56 (7.17-18.24)	9.69 (8.47-18.61)	10.01 (8.15-17.27)	9.97 (6.89-15.88)	10.64 (8.6-13.74)
Test statistic	U=36.5	t=-2.689	U=40	U=47	U=56.000	U=67.5	t=-2.959	t=-1.581
P-value	0.066	0.013	0.103	0.221	0.485	0.977	0.007	0.127

**Table-III:** Univariate and Multiple Logistic Regression Analysis of Factors Associated with Patient and Control Groups.

	Group		Univariate			Multiple	
Variable	Control	Patient	OR (95% CI)	P-value	OR (95% CI)		P-value
Sex							
Male	13 (38.2)	21 (61.8)	Reference				
Female	7 (63.6)	4 (36.4)	0.354 (0.086-1.449)	0.149	0.144 (0.009-2.428)		0.179
Years	58.05±7.19	63.08±10.93	1.06 (0.991-1.133)	0.089	0.944 (0.811-1.099)		0.458
ALT(U/L)	28.24±34.38	17.15±5.94	0.886 (0.785-1.001)	0.051	-		-
ALP(U/L)	64.31±15.06	150.55±141.88	1.076 (1.028-1.126)	0.002	-		-
Total bilirubin(mg/Dl)	0.64±0.19	4.8±19.21	15.653 (1.666-147.053)	0.016	-		-
Neutrophil(Bin/µL)	3.3±1.26	5.18±2.18	1.918 (1.203-3.058)	0.006	-		-
Lymphocyte(Bin/µL)	2.07±0.53	1.5±0.82	0.307 (0.116-0.814)	0.018	-		-
CRP(Miligram/litre - mg/L)	2.78±0.99	7.33±4.44	2.48 (1.302-4.727)	0.006	-		-
Neutrophil/lymphocyte ratio( N/L Ratio)	1.99±0.64	6.44±8.67	2.475 (1.16-5.28)	0.019	-		-
Platelet/lymphocyte ratio( P/L Ratio)	102.47±28.23	177.77±100.17	1.022 (1.005-1.039)	0.012	-		-
Hsa-miR-200c-3p	6.48±1.46	9.03±2.58	1.942 (1.261-2.99)	0.003	2.218 (0.96-5.127)		0.062
Hsa-miR-155-5p	7.79±1.59	134.99±436.29	3.202 (1.603-6.396)	0.001	-		-
Hsa-miR-20a-5p	7.7±1.62	145.9±472.17	2.456 (1.43-4.217)	0.001	42.642 (2.375-765.706)		0.011
Hsa-miR-221-3p	8.57±1.93	184.85±496.08	1.83 (1.141-2.934)	0.012			
Hsa-miR-34a-5p	8.6±1.75	136.19±439.19	1.99 (1.184-3.345)	0.009	0.056 (0.005-0.669)		0.023
Hsa-let-7b-5p	5.19±1.2	72.15±315.81	8.004 (1.986-32.265)	0.003	-		-
Hsa-miR-125b-5p	5.91±1.19	64.94±272.72	-	-	-		-

## DISCUSSION

Hepatocellular carcinoma (HCC) is the most common type of liver cancer and the second most common cause of cancer-related deaths.^3^ Recent studies have focused on investigating the signaling pathways involved in HCC progression and potential therapeutic targets. In particular, the overactivation of the Wnt/β-catenin pathway has been reported to play a significant role in the pathogenesis of HCC.^7^,^8^ In HCC, miRNAs act as external regulators of the Wnt/β-catenin pathway. miRNAs are small, non-coding RNAs that regulate gene expression at the transcriptional level.^9^

In our study, an increase in the expression of Hsa-miR-20a-5p, Hsa-let-7b-5p, and Hsa-miR-125b-5p was observed, indicating their potential to regulate oncogenic signaling pathways such as Wnt/β-catenin. The Wnt/β-catenin pathway is known to promote cancer cell proliferation, invasion, and metastasis. The involvement of these miRNAs in various steps of this pathway contributes to our understanding of their roles in cancer biology and highlights the potential discovery of novel molecular markers that could be targeted in the treatment of HCC. In this context, further research on how miRNAs affect Wnt pathways will deepen our understanding of HCC biology and contribute to the development of potential therapeutic strategies.

The miR-200 family plays a crucial role in regulating liver cancer development and presents new possibilities for HCC treatment. Wu et al.^10^ investigated the biological function of miR-200 and its target WNT BMI1. It was found that miR-200 directly targets BMI1 and suppresses tumor development by promoting apoptosis. Another study by Liu et al.^11^ reported a significant decrease in miR-200 levels in F344 rat HCC cells and demonstrated that miR-200a inhibits HCC development by targeting the β-catenin pathway.

In the present study, no significant difference was found in the expression of miR-200 between the HCC and control groups. However, significant differences were observed in the expression of miR-200 in relation to HBV positivity. Additionally, the expression levels of miR-155 did not show a significant difference between the HCC and control groups. High levels of miR-155 have been reported in HCV-infected patients.^12^ MiR-125 is positively associated with APC and plays a role in preventing hepatocarcinogenesis.^13^ MiR-125b inhibits EMT by targeting SMAD2-4.^14^ Studies by Gougelet et al.^15^ and Zhu et al.^16^ have shown that miR-34 is regulated by β-catenin and plays an important role in HCC. Moreover, it has been reported that let-7 inhibits the Wnt/β-catenin pathway, suppressing HCC cell proliferation.^17^ MiR-125 is positively associated with APC and plays a role in preventing hepatocarcinogenesis. MiR-125b inhibits EMT by targeting SMAD2-4 22. Studies by Gougelet et al. and Zhu et al.^15^,^16^ have shown that miR-34 is regulated by β-catenin and plays an important role in HCC. Furthermore, it has been reported that let-7 inhibits the Wnt/β-catenin pathway, suppressing HCC cell proliferation.

In the study conducted by Yi-Shan Tsai and colleagues, the potential of circulating Let-7 family microRNAs to predict HCC risk in CHC patients was investigated. The study found that the Let-7 family (except for let-7c) exhibited a negative correlation with fibrosis scores and that higher levels of let-7i were associated with a reduced risk of HCC development. However, the findings of Yi-Shan Tsai and colleagues need to be validated in different patient populations and larger cohorts.^18^

In this study, however, high expression levels of both miR-125 and let-7 were found in HCC tissues. These findings are consistent with previous studies suggesting that miR-125 and let-7 can inhibit the Wnt/β-catenin pathway and affect HCC development. Moreover, the association of miR-125 with APC and the potential of let-7 to suppress the Wnt/β-catenin pathway further highlight their critical roles in HCC.

In this study, routine blood tests were found to have predictive value in assessing the prognosis of HCC and monitoring treatment outcomes. The comparison of clinical data with miRNA expression revealed that some findings were consistent with the literature, while others showed discrepancies. Notably, the high expression of miR-20, miR-125, and let-7 molecules was found to be consistent with some studies in the literature, whereas the expression of miR-34 and miR-155 showed differences.

This study contributes to our understanding of the role of miRNAs in hepatocellular carcinoma (HCC) pathogenesis, particularly observing that miR-20a-5p, let-7b-5p, and miR-125b-5p influence Wnt/β-catenin signaling pathways, shaping HCC development. These findings align with the existing literature and offer a new perspective on HCC biology.

### Strenght & Limitation:

One of the strengths of our study is the use of a prospective case-control design, which has increased the reliability of the data and provided a solid foundation for clinical applications of the findings. Additionally, RNA was isolated from paraffin-embedded liver tissues, and miRNA expression levels were accurately measured. This allowed us to highlight the potential of miRNAs such as miR-20, miR-125, and let-7 as biomarkers in HCC. Future research could profile miRNAs in other types of liver cancer to test the universal applicability of these biomarkers. Our study focused solely on hepatocellular carcinoma (HCC) patients, investigating the potential role of various biomarkers, particularly miRNAs, in this cancer type. The results indicate that HCC significantly differs from other liver cancer types. Future studies aim to profile miRNAs in other types of liver cancer to test the universal applicability of these biomarkers.
